# Psychosocial aspects of obesity in adults with psoriasis: A systematic review

**DOI:** 10.1002/ski2.33

**Published:** 2021-05-03

**Authors:** N.T. Pavlova, K. Kioskli, C. Smith, F. Picariello, L. Rayner, R. Moss‐Morris

**Affiliations:** ^1^ Health Psychology Section, Psychology Department King's College London Institute of Psychiatry Psychology and Neuroscience London UK; ^2^ Department of Computer Science, Centre for Adaptive Computing Systems University of London London UK; ^3^ Gruppo Maggioli, Research and Development Lab Athens Greece; ^4^ St John's Institute of Dermatology Guy's and St Thomas' NHS Foundation Trust London UK; ^5^ Department of Psychological Medicine King's College London Institute of Psychiatry Psychology and Neuroscience London UK

**Keywords:** psoriasis, psychodermatology, quality of life

## Abstract

**Background:**

Excess weight is a common (30%–40%) multifactorial concern that remains understudied in adults with psoriasis.

**Objectives:**

This systematic review aimed to synthesise the evidence on the psychosocial factors associated with body weight in psoriasis and to use these findings to inform clinical practice. The review was registered with PROSPERO (registration number: CRD42020201138).

**Methods:**

Electronic databases, related reviews and associated reference lists were searched. Observational and experimental studies reporting on the relationship of psychosocial factors to weight‐related outcomes in adults with body mass index (BMI) of ≥30 kg/m^2^ and psoriasis were eligible. The methodological quality of the included studies was assessed using the Critical Appraisal Skills Programme (CASP).

**Results:**

Eighteen studies were included in the review, the majority of which (*n* = 16) examined cross‐sectional associations between psychosocial factors and weight outcomes. Although the strengths of the associations were heterogeneous, most studies confirmed the positive association between high BMI and increased reports of depression and anxiety, impaired quality of life, deteriorated sleep quality, sexual dysfunction, and daily functioning issues. Only four studies were rated as high quality.

**Conclusions:**

The current evidence of the association between psychosocial factors and weight‐related outcomes is largely cross‐sectional with unclear directionality of causality. Longitudinal studies are needed to examine the replicability and generalisability of the examined obesity‐related psychosocial factors in psoriasis. Theoretical exploration of subgroup differences and similarities may pave the way towards intervention personalisation, and ultimately, improved patient outcomes.

## Graphical abstract

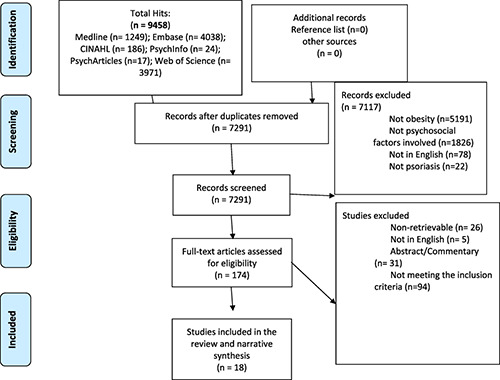

Obesity is a common yet multifactorial issue for people with psoriasis due to the unique psychological challenges which they face. This systematic review aimed to identify relevant psychosocial issues which are associated with excess weight to inform future intervention studies which aim to look beyond traditional weight loss approaches for weight loss such as low‐calorie diets.

1

**What is already known about this topic?**

- Maintenance of a healthy body weight offers the opportunity to reduce the significant burden of psoriasis and to improve treatment response
- Most therapeutic approaches are generic and emphasize on diet and physical activity as strategies to reduce weight in the context of psoriasis.
- Yet people with psoriasis face skin‐related issues such as embarrassment that may impede their abilities to commit to the recommended weight loss behaviours.

**What does this study add?**

- To unpack the positive association between excess weight and worsen mental health, an in‐depth understanding of patients' experiences of living with psoriasis and comorbid obesity is a priority.
- Future well‐designed prospective studies should recognize the complex and perhaps multifactorial nature of managing body weight in the context of psoriasis.
- The inclusion of tailored psychosocial support is likely to support healthy body weight management and ultimately patients' outcomes.

## 2 INTRODUCTION

Psoriasis and obesity are major public health issues, both on the rise.^1^, ^2^ Psoriasis is a debilitating inflammatory systemic disease, affecting an estimated 29.5 million adults worldwide and about 1 million in the UK population.^2^ A third of people living with psoriasis are obese, defined as a body mass index (BMI) of ≥30kg/m^2^.^3^ Obesity is a risk factor for psoriasis onset and exacerbation.^4^, ^5^ Patients with severe psoriasis are more likely to become obese than those with mild‐to‐moderate disease phenotypes (odd ratios of 1.55–3.05 and 1.17–1.82, respectively).^6^, ^7^ Similarly, obese psoriasis patients are less likely to respond to biologic therapy and systemic treatment than healthy weight patients^5^, ^8^, ^9^, ^10^

Obesity in psoriasis is multifactorial and is best understood through a biopsychosocial approach, including the role of genetics, epigenetic, behavioural and environmental factors.^11^ Since genetic factors are non‐modifiable, increasing physical activity and improving healthy dietary habits may positively affect obesity trends.^12^, ^13^ Yet people with psoriasis face psoriasis‐associated barriers such as embarrassment about their skin condition which may affect their body image negatively, impede physical activity behaviours and affect their self‐esteem to live healthily.^14^ Lifestyle interventions for obese people with psoriasis must address these challenges since reducing the activity of inflammatory disease such as obesity could improve both psoriasis and related comorbidities.^19^

Tailored illness‐specific weight‐loss interventions that address psychosocial difficulties, including emotional and behavioural issues, provide a more balanced approach to weight loss.^15^, ^16^ Thus, they may be more effective in supporting weight loss than generic ones and ultimately lead to better weight loss and psychological outcomes.^17^, ^18^

A recent systematic review highlighted that none of the weight‐loss interventions in psoriasis included consideration of psychological or social factors.^19^ Instead, general models of weight loss such as low‐calorie interventions embedded in pharmacological treatments were used.^20^, ^21^, ^22^, ^23^, ^24^ It has been shown that weight loss can lead to improved psoriasis severity in addition to the effects of systemic treatment in the short term, however, weight is typically regained in the long run. This may be due to a failure to address the complex and multifactorial aetiology of obesity observed in the general population^25^, ^26^ yet understudied in psoriasis. Weight loss interventions that look beyond dietary changes and exercise plans are needed. This is aligned with the National Institute for Health and Care Excellence^27^ guidelines on psoriasis stipulating the importance of prioritising the patient's psychological well‐being in any treatment regimen.

**TABLE 1:** Inclusion and exclusion criteria

Criteria	Inclusion criteria
Population	Adults (18 years or older) with diagnosis of psoriasis
Exposure, comparator and outcomes	o Changes in weight in relation to psychosocial factors
	o Psychosocial themes in the context of weight
Study type	o Observational studies
	o Randomised control trials (RCT) if matching the outcome requirements
	o Qualitative studies

In summary, maintenance of healthy body weight offers the opportunity to reduce the significant burden of psoriasis. To date, most therapeutic approaches fail to consider the psychosocial factors relevant to weight outcomes in psoriasis.^20^, ^21^, ^22^, ^23^ An evidence‐based understanding of the specific psychosocial factors implicated in weight in psoriasis might pave the way towards more tailored and effective interventions that address the complex needs of patients with psoriasis, and ultimately, improved patients' outcomes.

This review, therefore, is aimed to (i) identify psychosocial factors related to body weight in people with psoriasis and (ii) quantify the magnitude of their relationship by calculating effect sizes and (iii) identify psychosocial themes from qualitative studies that discuss psoriasis in the context of body weight.

## 3 MATERIALS AND METHODS

### 3.1 Literature search

This systematic review was registered with PROSPERO (registration number: CRD42020201138) and was conducted according to the PRISMA guidelines.^28^ A literature search was performed in October 2020 using the following databases: PsycInfo, PsychArticles, Medline, Embase, Cinahl, Web of Science and Scopus. A combination of weight‐related, psychosocial and psoriasis terms were tailored to each database (Appendix S1). Secondary searching of bibliographies was also performed. The search was limited to full‐text articles in English to allow for adequate appraisal of the findings.

### 3.2 Selection criteria

The inclusion and exclusion criteria were specified using the PECOS approach (Table 1). The selection of relevant articles was performed by two authors (NP and KK).

### 3.3 Data extraction and quality assessment

PRISMA guidelines^29^ guided the predefined criteria for data extraction Table 1 that was conducted independently by two authors (NP and KK). The extracted information included: study design, number of participants, characteristics of the patient sample (age, mean BMI, psoriasis severity and duration), comparator group, type of (correlate) psychosocial measure, weight‐related data such as BMI and/or waist circumference (outcome) and key findings.

**TABLE 2:** Summary of the studies' characteristics

Author(s) name, date, location, study design	Number of participants, Proportion male (%)	Mean age (SD or range)	Mean BMI (SD); %obese	Mean PASI (SD or range), psoriasis duration (SD)
Cohen et al., 2015, US, cross‐sectional^34^	*N* = 351, 48%	50.9 (17.3)	29.6 (6.7); NR	NR, NR
Tabolli et al., 2012, Italy, RCT^39^	*N* = 202, 61.4%	47.94 (15.02)	27.26 (5.88), at baseline; NR	23.16 (12.4), NR
Innamorati et al., 2016, Italy, cross‐sectional^35^	*N* = 197, 51%	50.45 (15.24)	27.4 (5.94); 24%	3.90 (3.03), 18.24 (13.46)
	
Grozdev et al., 2012, USA, cross‐sectional^45^	*N* = 429, 54%	48.7 (15.4)	30.4 (7.5); NR	23.16 (12.4), NR

Crosta et al., 2014, Italy, cross‐sectional^60^	*N* = 100, 51%	47.7 (12.9)	NR; 19%	4 (5.6), 13.8 (12.7)
	
Sacmaci and Gurel, 2019, NR, cross‐sectional^55^	*N* = 60, 50%	42.8 (13.1)	28.5 (4.7); NR	10.1 (9.7), 11.8 (8.1)

Cakmur and Dervis, 2015, Turkey, cross‐sectional^42^	*N* = 100, 50%	38.63 (NR)	NR; 46%, overweight	8.4 (NR), 15.05 (10.36)

Storer, Danesh, Sandhu, Pascoe, Kimball, 2018, USA, cross‐sectional^47^	*N* = 47, 55%	51 (16)	NR; 100% obese	8.8 (8.2), NR

Bronckers et al., 2016, Netherlands, cross‐sectional^43^	*N* = 75, 29.3%	21.0 (8.0)	23.4 (6.9); NR	4.4 (4.9), NR

Barrea et al., 2016, Italy, case‐control^46^	*N* = 180, 71%	50 (21.0–65.0)	30.2 (6.1); 47.2%	6 (0.2–28.8), NR
Adawiyah, Moonyza, Hatta, Rizal, Felix, 2017, Malaysia, cross‐sectional^53^	*N* = 79, 0%	40.32 (10.04)	28.3 (5.51); NR	8.4 (0.1–34.5), N

Molina‐Leyva et al., 2013, Spain, cross‐sectional^54^	*N* = 80, 50%	43.4 (12.7)	28.2 (6.7); NR	2.7 (1.2–7.1), NR

Remrod, 2013, Sweden, cross‐sectional^56^	*N* = 101, 55%	43.5 (13.8)	26.2 (4.5); NR	5.4 (4.3), NR

Lewinson et al., 2017, Italy, retrospective^40^	*N* = 73 447, 51%	NR (20–90)	NR; 18.26%	>95% mild psoriasis
	
Kim et al., 2014, USA, cross‐sectional^38^	*N* = 114, 69%	47.7 (NR)	NR; 29.8%	10.2 (NR), 21.3 (NR)
	
Tang et al., 2013, Malaysia, cross‐sectional^44^	*N* = 250, 54%	42.5 (18–83)	26.9 (5.7); 26.8%	9.9 (0.2–69.2), 10 (0.5–49)
	
Ryan et al., 2013, Dallas, Texas, and Dublin, cross‐sectional^36^	*N* = 354, 57.6%	48 (18–78)	NR; NR	4.3 (0–44.8), NR

Sanchez‐Carazo et al., 2014, Spain, cross‐sectional^41^	*N* = 1022, 60%	NR; 26%	NR; 26%	61%, mild‐to‐moderate, NR
		

Most studies (*n* = 16) used the Psoriasis Area and Severity Index (PASI)^30^ as a measure of disease severity but used different cut‐offs. To achieve consistency, we defined psoriasis as mild‐to‐moderate (PASI<10) and severe (PASI≥10) based on the extracted PASI data and recommended cut‐offs.^31^

BMI categories were defined consistently in all studies as normal weight (BMI ≤ 25 kg/m^2^), overweight (BMI 25 to 29.9 kg/m^2^) and obese (BMI ≥ 30 kg/m^2^).

The methodological quality of the studies was independently assessed by two authors (NP, KK). In event of disagreement, the authors would discuss the matter and if a solution was not found the opinion of the third author (RMM) would be sought. The quality of observational evidence and the baseline data from the randomised controlled trial were assessed using the Critical Appraisal Skills Program^32^ (Table S2; supporting information).

### 3.4 Data analysis

The included studies used different methods to analyse their data. To make meaningful summaries of the effects across them, we calculated the magnitude of associations from correlational methods when enough data (Mean and SD) were available and it was defined as weak (*d ≤ *0.2), small (*d* = 0.2), medium (*d* = 0.5) or large (*d* = 0.8).^33–34^ Table S3 When data were not available, the authors of the eligible papers were contacted for further information.

## 4 RESULTS

### 4.1 Study selection and characteristics

**FIGURE 1:**
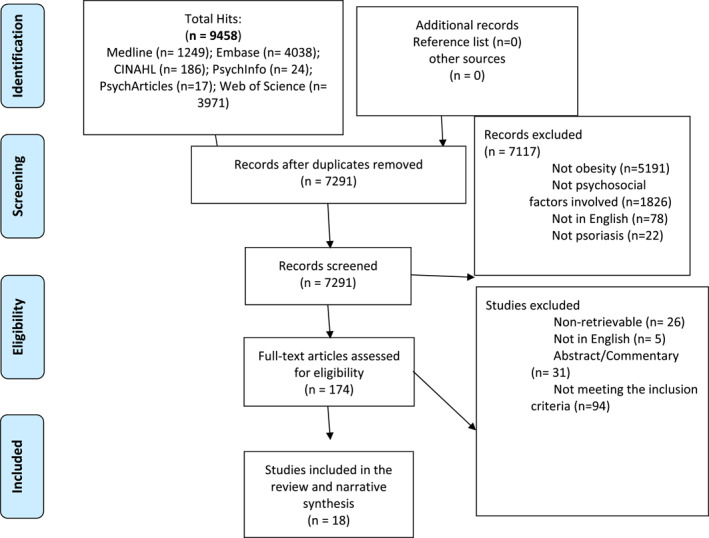
Flowchart‐Selection process

A flow diagram for the inclusion of studies is depicted in Figure 1. Eighteen studies met the inclusion criteria: one RCT exploring baseline associations, 16 cross‐sectional and one retrospective study.

The study characteristics are summarized in Table 2 below. The mean BMI in the included studies was ≤30 kg/m^2^ (*n* = 16) and mostly considered as a confounding variable in their analyses. Most studies' (*n* = 14) samples consisted of participants with mild‐to‐moderate psoriasis. Table S1 (supporting information) summarizes the relationship of psychosocial factors to weight‐related outcomes by psoriasis severity.

To date, most studies have focussed on the relationship between weight‐related outcomes and depression, and quality of life (QoL) among psoriasis patients (*n* = 12), using BMI (*n* = 18) and waist circumference (*n* = 1) as outcome measures. Anxiety, sexual dysfunction, lifestyle factors, personality traits, beliefs and emotional regulation were less frequently examined factors in relation to weight outcomes.

### 4.2 Quality assessment

The quality of the studies (*n* = 18) was assessed using CASP.^32^ Four studies were rated as high quality, 10 as moderate and four as low (Table S2; supporting information). The psychosocial variables included in each study and the calculated magnitude of the relationship between psychosocial factors to weight‐related outcomes is available in Table 2 and discussed under the psychosocial sub‐groupings below.

### 4.3 Depression and anxiety

Six studies examined the association between BMI and depression, in addition to their main objectives: four cross‐sectional^35^, ^36^, ^37^, ^38^ one RCT^39^ examining the baseline correlation, and one retrospective population‐based study.^40^ Four out of the six studies found that patients with higher BMI reported significantly greater levels of depression.^35^, ^36^, ^37^, ^39^

Effect sizes could be calculated for two of these studies. One showed a weak effect for the correlation between BMI and depression^39^ and the other showed a large between‐groups effect for the comparison between normal weight and obese patients.^37^ Differences in the magnitude of the effect sizes can be attributed to the different ways used to approach the data‐correlational versus group differences.

Of the two disparate studies, one reported comparable levels of depression between normal weight and obese patients.^38^ However, unlike the four studies above which used validated measures of depression, this study assessed depression with a single question, and the sample size was small. The other disparate study was a retrospective‐population study reporting that non‐obese people are more likely to be depressed than obese people.^40^ Missing data were more prevalent in the depressed than the non‐depressed group which somewhat undermines the association. The association between BMI and anxiety was explored in two studies.^35^, ^39^ Both reported a significant positive association between higher BMI and increased anxiety.

### 4.4 Quality of life (QoL)

Nine cross‐sectional studies^35^, ^36^, ^41^, ^42^, ^43^, ^44^, ^45^, ^46^ and one RCT^39^ examined the baseline association between BMI/obesity and QoL, in addition to their main objectives. Of these, seven studies reported negative associations between BMI and QoL,^35^, ^39^, ^42^, ^43^, ^45^, ^46^, ^47^ and waist circumference and QoL,^46^ indicating that higher BMI and waist circumference are linked to a worse QoL. The magnitude of the associations was estimated to be weak to medium in most studies^42^, ^43^, ^45^, ^46^, ^47^ and large using the RCT's baseline data.^39^ The RCT measured QoL used Skindex‐29,^39^ while the cross‐sectional studies used the Dermatology Quality of Life Index (DLQI).^35^, ^42^, ^43^, ^46^, ^47^ Skindex‐29 includes a wider range of emotional items which are addressed by a single item in the DLQI.^48^ Similar findings were observed for QoL in one study where obese people with psoriasis were significantly more likely to report worse QoL than obese people without psoriasis.^35^ These findings differed in the rest of the cross‐sectional studies which reported no association between BMI and QoL.^36^, ^41^, ^44^ Two of these studies^36^, ^44^ had much larger cohorts of participants than the ones reporting a positive association.^35^, ^39^, ^42^, ^43^, ^45^, ^46^, ^47^

Contrary to the above patterns, three of the studies that looked at mental health‐related QoL, as opposed to general QoL, found that higher BMI was associated with better mental health‐related QoL.^35^, ^39^, ^45^ One showed a weak effect,^45^ reporting a positive association^36^ and a large effect.^39^

### 4.5 Sexual dysfunction

Four cross‐sectional studies^36^, ^38^, ^53^, ^54^ investigated the association between BMI and sexual dysfunction, in addition to their main objectives. Of these, three^36^, ^53^, ^54^ reported a positive association between BMI and sexual dysfunction. The magnitude of the association was calculated as small^53^, ^55^ and large.^54^ Conversely, Kim et al.^38^ reported that the sexual functioning of obese and normal‐weight patients with psoriasis was comparable. However, unlike the other studies, Kim et al.^38^ used a non‐validated measure of sexual functioning.

### 4.6 Lifestyle factors

Two cross‐sectional studies^37^, ^38^ looked at the association between BMI and lifestyle‐related factors, in addition to their main objectives, and reported medium positive associations between BMI and difficulty in working, taking care of things at home, and getting along with people.^37^ Obese people with psoriasis were significantly more likely to avoid common physical activities such as swimming and are more likely to use recreational drugs than normal‐weight people with psoriasis.^38^

Furthermore, two cross‐sectional studies^38^, ^53^ were consistent in finding a high BMI was associated with more subjective sleep problems. One showed a medium positive association^53^ and the other reported that obese people with psoriasis had significantly more sleep problems than normal‐weight people with psoriasis.^53^

### 4.7 Personality factors, beliefs and emotional regulation

Four cross‐sectional studies^35^, ^38^, ^41^, ^56^ looked into the association between BMI in psoriasis and personality factors, beliefs or emotional regulation, in addition to their main objectives. Of these, one study^56^ assessed the relationship between BMI and embitterment, trait irritability, mistrust and verbal aggression. The study reported weak positive correlations between BMI and these personality traits, except for embitterment where the association was small and negative.^56^ Two of the other studies compared obese people versus healthy weight people with psoriasis.^37^, ^41^ Kim et al. (2014)^38^ found that obese people with psoriasis had a greater need to hide psoriasis, reported stronger beliefs that psoriasis caused weight gain, had lower self‐confidence and were more likely to perceive that weight is a problem in managing psoriasis than normal weight patients.^38^ Similarly, medium positive was obtained between‐group differences for higher interoceptive awareness and bulimia, and small positive between‐groups differences for higher interpersonal distrust and higher ineffectiveness; all these factors were more common in obese people with psoriasis versus those with normal weight.^41^

One additional cross‐sectional study^36^ looked at the differences between obese people with and without psoriasis and reported that obese people with psoriasis were significantly more likely to have severe alexithymia, difficulties in emotional regulation, and food cravings versus those without psoriasis.

## 5 DISCUSSION

This systematic review included 18 studies to examine the association between psychosocial factors and body weight in people with psoriasis. It was largely agreed that increasing weight is associated with higher rates of depression, anxiety, and poorer QoL. A small number of studies also showed a positive association between higher weight and poor subjective sleep quality, sexual dysfunction, and emotional regulation issues. The included studies were all cross‐sectional. This precluded determining the direction of causality of the examined associations and highlighted the surprising lack of prospective studies. The understudied role of weight‐specific cognitions such as self‐confidence to engage in healthy behaviours and body image issues that are likely to affect the success of committing to weight‐loss behaviours was also observed.^37^, ^52^ One of the reviewed studies showed that beliefs that psoriasis caused weight gain was related to higher BMI.^37^ Such beliefs may result in a fatalistic response to weight management and need to be explored further.

The findings from this systematic review are consistent with the literature in the general population which suggests that the rates of depression, anxiety and impaired QoL are significantly higher among obese individuals than those with normal weight.^49^, ^50^, ^57^, ^58^ The mostly weak to small magnitude of the associations between weight outcomes and psychosocial factors in psoriasis can be explained by several methodological limitations. Males were over‐represented in most of the included studies.^35^, ^38^, ^39^, ^41^, ^46^ There are well‐documented gender discrepancies in the general obese population suggesting a significant association between obesity and poor mental health in females, but not males (51, 59). Luppino et al.^50^ demonstrated that it is almost a double risk for women with obesity to develop depression in comparison with men (67% and 31%, respectively). The included studies did not acknowledge these gender differences and no gender‐stratified results were available. The majority of the included patients (*n* = 13/18) had mild‐to‐moderate psoriasis.^35^, ^36^, ^40^, ^41^, ^42^, ^43^, ^44^, ^46^, ^47^, ^53^, ^54^, ^56^, ^60^ This limits the conclusions about the relationship between mental health and weight as obesity is more prevalent among patients with severe psoriasis phenotypes than milder ones.^19^

Furthermore, the included studies did not investigate the curvilinear relationship between BMI and the risk of depression and poor mental health that has been demonstrated in the general population; where underweight (BMI <18.5 kg/m^2^) and severely obese (BMI of ≥40 kg/m^2^) are the most strongly correlated with depression and poor mental health.^50^, ^51^, ^59^, ^61^ Most studies (*n* = 17/18) looked at BMI as a continuous variable and the average BMI of their samples fall in the overweight category. The studies that dichotomised participants into non‐obese and obese used the cut‐off points with very close proximity. Thus, individuals close to but on the opposite side of the cut‐off point are characterised as being very different rather than very similar. Excess body weight in all studies was based on BMI which does not completely reflect trends in body fat.^62^ Whole‐body fat mass, but not whole‐body non‐fat mass, causes depression.^63^ Therefore, more sensitive tools for measuring adiposity such as waist circumference are needed to determine the strength of the association with psychosocial factors.

Beyond the role of depression, anxiety, and QoL, cognitive factors such as beliefs, thinking styles, and body‐image issues were largely overlooked. Approaches that address cognitions can outperform the outcomes achievable by traditional lifestyle‐modification weight‐loss treatments.^64^ Since people with obesity and psoriasis are likely to have low self‐confidence and body image issues particularly related to their skin condition, these can be important processes in maintaining unhealthy weight through behavioural withdrawal and inactivity.^37^ Yet, so far only traditional approaches have been used in psoriasis that overlook patients' weight‐specific beliefs.^19^ One reviewed study suggested that addressing beliefs about the relationship between psoriasis and weight may also be important.^37^ Thus, future research should combine traditional behavioural techniques aimed at facilitating weight loss with cognitive techniques such as addressing negative body image to reduce weight‐loss treatment attrition and increase weight loss and weight maintenance behaviours.^59^, ^65^

### 5.1 Limitations

The review was limited to adults and findings cannot be generalised to paediatric populations. There was variability in measures of psychosocial factors which limits comparisons between studies. The focus on bivariate analyses and dichotomised multiple between‐group analyses to facilitate comparison across studies may have limited an in‐depth understanding of psychosocial factors based on multivariable models and more subtle subgroup analyses. Given the exclusively cross‐sectional nature of the evidence depression anxiety, and impaired QoL are likely to also be consequences of excess weight.

## 6 CONCLUSION

This systematic review highlighted the multifaceted nature of excess weight in psoriasis and its positive association with worsening psychosocial outcomes that should be addressed as a part of tailored weight‐loss treatment approaches. Future studies should include the following^1^: explore gender subgroup differences and present stratified results^2^; conduct well‐designed longitudinal cohort studies which include people with severe psoriasis to test the relationships between psychosocial factors and weight outcomes, ideally using more body fat‐sensitive measures such as waist circumference^3^; conduct qualitative research to explore the understudied experiences of weight management of obese patients with psoriasis^4^; use theory‐based approaches to select psychosocial factors associated with obesity to building a better understanding of obesity in the context of psoriasis, subsequently leading to theory‐ and evidence‐based tailored interventions that are likely to achieve better and lasting clinical outcomes.

## CONFLICT OF INTEREST

The authors declare they no conflicts of interest.

## Data Availability

The data that supports the findings of this study are available in the supplementary material of this article.
